# Allelopathic activity of *Phragmites australis* against *Bolboschoenus planiculmis* and the involved active allelochemicals

**DOI:** 10.3389/fpls.2025.1607628

**Published:** 2025-06-23

**Authors:** Liu Yang, Chong Chen, Yong Wang, Jingyao Wang, Fengxue Shi, Keming Yue, Xue Wang, Chunguang He

**Affiliations:** ^1^ Key Laboratory of Wetland Ecology and Vegetation Restoration, Ministry of Ecology and Environment, Northeast Normal University, Changchun, China; ^2^ Jilin Provincial Natural History Museum, Northeast Normal University, Changchun, China

**Keywords:** allelopathy, phenolic allelochemicals, aqueous extract, germination, seedling growth, HPLC-MS/MS

## Abstract

**Introduction:**

*Bolboschoenus planiculmis* (F. Schmidt) T. V. Egorova plays an important ecological role in wetland ecosystems by providing essential habitat and food resources for the critically endangered Siberian crane (*Grus leucogeranus*). It frequently coexists with *Phragmites australis* (Cav.) Trin. ex Steud. (reed) in natural wetland communities; however, the allelopathic activity of reed on *B. planiculmis* remains poorly understood.

**Methods:**

This study investigated the allelopathic effects of reed on *B. planiculmis* and identified the phenolic allelochemicals involved. Aqueous extracts from individual reed organs (roots, stems, and leaves), as well as from a mixture of these organs in equal mass proportions, were prepared at two concentrations (7% and 14%) using plant materials collected during both the nutrient and reproductive growth stages.

**Results and discussion:**

Pot experiments revealed that reed aqueous extracts exhibited significant inhibitory activity on the germination and seedling growth of *B. planiculmis*. The leaf extract showed relatively stronger inhibitory effects compared to the extracts of other organs, especially in the nutrient growth stage. A total of 24 phenolic compounds, including 13 phenolic acids, 9 flavonoids, and 2 coumarins, were identified as potential allelochemicals in reed aqueous extracts. The concentration of phenolic allelochemicals in leaf extract was much higher than that in root and stem extracts. These findings demonstrate the allelopathic inhibitory effect of reed on the germination and seedling growth of *B. planiculmis*, primarily mediated by active phenolic compounds derived from leaves. Notably, this study is the first to identify flavonoids and coumarins, in addition to phenolic acids, as potential allelochemicals contributing to the allelopathic effects of reed on *B. planiculmis* in wetland ecosystems. This study enhances our understanding of ecological interactions among wetland plants and provides guidance for the conservation and management of the key functional species *B. planiculmis*.

## Graphical Abstract

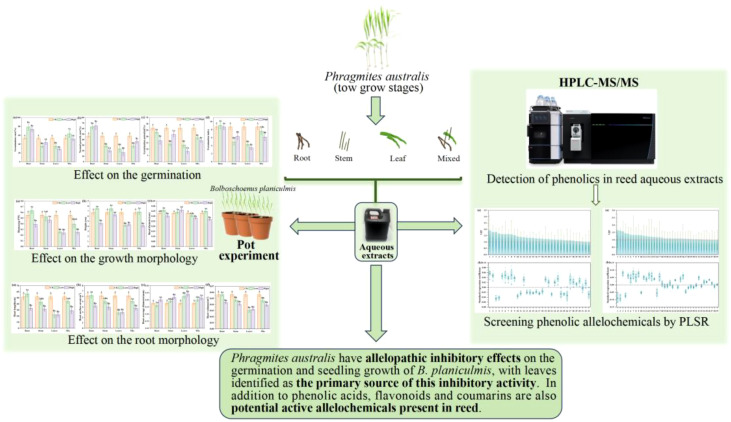

## 1 Introduction

*Phragmites australis* (Cav.) Trin. ex Steud. (reed) is one of the most globally widespread wetland plants, found on every continent except Antarctica (Packer et al., 2017). Under favorable conditions, it typically forms dense monospecific stands, and its expansion or invasion can significantly alter ecosystem structure and function (Wails et al., 2021). Allelopathy is a significant mechanism of plant-plant interaction, involving the inhibition of neighboring plant growth through the production and release of secondary metabolites, commonly known as allelochemicals (Hierro and Callaway, 2021). This mechanism is considered an important driver of the reed’s strong interspecific competitive ability and broad distribution (Uddin et al., 2014b, 2017). In southeastern Australia, wetland vegetation dominated by *Melaleuca ericifolia* is threatened by the expansion of reed, as allelochemicals secreted by its roots can inhibit both germination and growth of *M. ericifolia* (Uddin et al., 2014b). Similarly, in the coexisting communities of reed and *Suaeda salsa* in China’s Yellow River Delta, allelopathy serves as a crucial ecological adaptation mechanism that enables reed to maintain a strong interspecific competitive advantage (Gao et al., 2022). Aqueous extracts of reed have been shown to significantly inhibit the germination and seedling growth of *S.salsa* (Gao et al., 2022).

The Momoge National Nature Reserve serves as a critical stopover site along the migratory route of the critically endangered Siberian Crane (*Grus leucogeranus*) (Wang Y. et al., 2022). *Bolboschoenus planiculmis* (F. Schmidt) T. V. Egorova is a key protected plant species within the reserve, with its communities providing primary stopover habitat and its underground tubers serving as an essential food source for Siberian Cranes (An et al., 2021). In recent years, however, *B. planiculmis* has undergone population decline and a notable reduction in distribution area, posing a serious threat to the cranes’ stopover and survival (An et al., 2018; Zhang et al., 2022). Within the reserve, *B. planiculmis* frequently co-occurs with reed in the same plant communities, but it exhibits poor growth performance and shows a trend of being gradually displaced by reed (Song et al., 2015; Ding et al., 2021; Tang et al., 2024; Liu et al., 2018). Nevertheless, whether reed influences the growth of *B. planiculmis* through allelopathic interactions remains uncertain.

The inhibitory allelopathic effects of reed on seed germination and seedling growth have been documented in a variety of plant species, including model species such as *Lactuca sativa* and *Arabidopsis thaliana*, economic crops such as *Vigna radiata* and *Nicotiana tabacum* (Rudrappa et al., 2007), and common weeds like *Poa labillardierei* and *Avena fatua* (Rudrappa et al., 2007; Uddin et al., 2017, 2014a, 2014b). Moreover, the allelopathic activity of reed appears to vary among its different organs. For instance (Gao et al., 2022) found that the underground parts (rhizomes and roots) exhibit stronger phytotoxic effects than the aboveground parts (stems and leaves), whereas (Uddin et al., 2012) reported that the leaves possess greater allelopathic inhibition activity than the rhizomes and roots. However, allelopathic effects are known to exhibit species-specific variability (Zhang et al., 2021). To date, the allelopathic effects of reed on the germination and growth of *B. planiculmis*, as well as the organ-specific variations in allelopathic activity, remain largely unexplored.

Phenolic compounds represent one of the most common classes of plant allelochemicals, encompassing phenolic acids, flavonoids, and coumarins (Macías et al., 2019). Reed synthesizes and releases phenolic compounds into its surrounding environment (Uddin and Robinson, 2017b), and the concentration of soluble phenolics in soils within reed-colonized areas has been reported to be more than twice that in non-colonized areas (Uddin et al., 2017). Gallic acid is a key allelopathic compound found in the root exudates of reed, capable of inhibiting root growth in plants (Rudrappa et al., 2007). Subsequent studies have also detected gallic acid in other reed tissues, including the leaves, flowers, and stems (Uddin et al., 2014). Moreover, the decomposition of reed litter releases a range of phenolic acids—such as p-coumaric acid, vanillic acid, sinapic acid, syringic acid, caffeic acid, protocatechuic acid, and gallic acid—that have been shown to inhibit algal growth (Nakai et al., 2006). In other Poaceae species, flavonoids also function as important allelochemicals (Anwar et al., 2021; Alghamdi et al., 2022; Vieites-Álvarez et al., 2023). For example, aqueous extracts of the invasive grass *Imperata cylindrica* contain compounds such as 5-methoxyflavone and 5,2′-dimethoxyflavone, which effectively suppress the growth of various weed species (Suzuki et al., 2018). However, flavonoids and coumarins with allelopathic activity have not yet been identified in reed.

This study aims to investigate the allelopathic effects of reed on *B. planiculmis* and to identify the active allelochemicals involved. Through pot experiments, we assessed the allelopathic effects of aqueous extracts derived from different reed organs and growth stages on the germination and seedling growth of *B. planiculmis*. In addition, we screened and identified potential phenolic allelochemicals in the reed extracts. We hypothesize that (1) aqueous extracts of reed inhibit the germination and seedling growth of *B. planiculmis*, with the inhibitory strength varying depending on the reed organ and growth stage; and (2) phenolic acids, flavonoids, and coumarins present in the extracts collectively contribute to the allelopathic effects of reed on *B. planiculmis*. The findings of this study will enhance our understanding of the ecological interactions between reed and *B. planiculmis*.

## 2 Materials and methods

### 2.1 Collection of experimental materials

The materials required for the experiment were collected from the buffer zone of the Momoge National Nature Reserve (123°46’98”E, 46°00’73”N). Whole reed plants were collected separately in early June 2023 during the nutrient growth stage and in late August 2023 during the reproductive growth stage. The whole reed plants were separated into roots, stems, and leaves, washed with tap water, then rinsed several times with ultrapure water, air-dried in the shade, and cut into 2 cm segments. Corms and *in-situ* soil (0–30 cm) of *B. planiculmis* were collected in early April 2023. Harvested corms were rinsed with tap water, disinfected in 5% sodium hypochlorite for 10 minutes, thoroughly washed with ultrapure water, blotted dry with absorbent paper, and stored at 4°C. Soil was air-dried, sieved through a 2 mm mesh, and homogenized to serve as the growth substrate for *B. planiculmis* in the pot experiment. Species identification was confirmed by Professor Yong Wang, and voucher specimens were deposited at the Natural History Museum of Northeast Normal University under registration numbers 1-001336-0010 (reed) and 1-001335-0008 (*B. planiculmis*).

### 2.2 Preparation of reed aqueous extracts

Based on previous literature (Khan et al., 2016; Wang C. et al., 2022) and preliminary experiments (**Supplementary Table S1**), concentrations of 7% and 14% were selected for the reed aqueous extracts. Each reed organ (140 g) was placed in a sterilized black bucket with 1 L of ultrapure water and soaked at 25°C for 48 hours, with ultrasonic treatment for 30 minutes every 12 hours to obtain crude extracts from the reed organs. The extract was then filtered and purified using 0.45 μm filter paper, resulting in a 14% (m/v) aqueous extract of reed roots, stems, and leaves. The roots, stems, and leaves were mixed in equal mass proportions and extracted using the aforementioned method to obtain the mixed aqueous extract. This extract simulated the natural coexistence of reed organs and their combined allelopathic potential. A portion of the prepared extract was stored in a 4°C refrigerator for pot experiments and diluted to a 7% concentration when needed. Another portion was stored in a –80°C freezer for the identification of phenolic allelochemicals.

### 2.3 Pot experiment

Pot experiments were conducted to investigate the allelopathic effects of reed from both the nutrient growth and reproductive growth stages on *B. planiculmis*. Eight different aqueous extracts (four organs × two concentrations) were prepared from reed for each growth stage. 0.9 kg *in-situ* soil of *B. planiculmis* was added to each pot (diameter: 18.5 cm, height: 8.8 cm), and the soil was moistened with 200 mL of ultrapure water. Fifteen healthy, similarly sized corms of *B. planiculmis* were evenly buried in the soil at the same depth. The pots were placed in an incubator set to 25°C, 60% humidity, and a 12 h light/12 h dark cycle with 250 μmol m^−2^ s^−1^ photosynthetic photon flux density during the light period for 10 days, during which the reed aqueous extract treatment was applied (**Table 1**).

**Table 1:** The reed aqueous extract treatments in the pot experiment.

Reed aqueous extracts			Treatment method
Growth stage	Organ	Concentration	
nutrient growth stage/reproductive growth stage	root	Low: 7%	Root extract (100 mL) and ultrapure water (100 mL) were applied on alternate days.
		High: 14%	
	stem	Low: 7%	Stem extract (100 mL) and ultrapure water (100 mL) were applied on alternate days.
		High: 14%	
	leaf	Low: 7%	Leave extract (100 mL) and ultrapure water (100 mL) were applied on alternate days.
		High: 14%	
	mixed	Low: 7%	Mixed extract (100 mL) and ultrapure water (100 mL) were applied on alternate days.
		High: 14%	

Each treatment consisted of four replicates, with each pot serving as an individual replicate. A daily addition of 100 mL ultrapure water was used as the blank control (CK).

The germination status was recorded daily. The following formulas were used to calculate the germination indicators: germination rate (GR), normal growth rate (NR), germination potential (GP), and germination index (GI):


(1) GR (%)=nN×100

(2) NR (%)=njN×100

(3) GP (%)=nkN×100

(4) $$\text{GI}=\sum G_{t}/D_{t}$$

where *n* is the number of germinated corms within 10 days, *n_j_* is the number of corms that germinated and grew normally (seedlings with true leaves and complete roots) within 10 days, *nk* is the number of germinated corms within the first 4 days, *Gt* is the number of germinated corms within *t* days, *Dt* is the corresponding number of germination days, and *N* is the total number of corms tested.

After 10 days, the growth parameters of the seedlings were measured, including biomass (fresh weight), plant height, and basal diameter. Root morphological parameters, including root length, root surface area, average root diameter, and root volume, were scanned using an Epson Perfection V850 Pro and analyzed with WinRHIZO 2017. The values for these indicators were obtained by calculating the average of all seedlings in each pot.

### 2.4 Detection of phenolic compounds in reed aqueous extracts

Phenolic compounds in the reed aqueous extract (high-concentration: 14%) were detected using high-performance liquid chromatography coupled with tandem mass spectrometry (HPLC-MS/MS). The reed extracts (100 µL) were placed in EP tubes and resuspended in pre-chilled 80% methanol by vortexing. The supernatants were collected following the standard procedure from Novogene (Deng et al., 2023). Then, the supernatant was injected into the HPLC-MS/MS analyzer using a Vanquish HPLC system (ThermoFisher, Germany) coupled with an Orbitrap Q Exactive HF mass spectrometer (Thermo Fisher, Germany) at Novogene Co., Ltd. (Beijing, China). In positive ion mode, mobile phase A was a 0.1% formic acid solution, while in negative ion mode, mobile phase A was a 5 mmol/L ammonium acetate solution. Mobile phase B was methanol, and the flow rate was 0.2 mL/min. The mobile phase gradient was set as follows: 0–2% B, 0–1.5 min; 2–85% B, 1.5–3 min; 85–100% B, 3–10 min;100–2% B, 10–10.1 min; 2% B, 10.1–12 min. The Q Exactive™ HF mass spectrometer was operated in both positive and negative polarity modes.

The raw data files were processed using Compound Discoverer 3.1 (ThermoFisher). The peak intensities were normalized to the total spectral intensity and used to predict the molecular formula based on additive ions, molecular ion peaks, and fragment ions. The peaks were matched to the mzCloud, mzVault, and MassList databases to obtain accurate qualitative and relative quantitative results.

### 2.5 Screening of reed phenolic allelochemicals

Partial Least Squares Regression (PLSR) analysis can address collinearity and noise among variables, and it is suitable for the complex analysis of small sample sizes and multiple variables (Wold et al., 2001). It has also been used in the screening of allelopathic substances (Reiss et al., 2018; Niazipoor et al., 2024). PLSR analysis was performed to investigate the relationship between the independent variable, “relative content of 91 phenolic compounds in reed aqueous extract,” and the dependent variable, “germination and seedling growth characteristics of *B. planiculmis*,” and screen the potential phenolic allelochemicals (Zhang et al., 2020). The dependent variables were divided into three groups based on their correlations, and each group was modeled separately (Wold et al., 2001). Model 1 focused on four germination indicators (GR, NR, GP, and GI), Model 2 on three growth indicators (biomass, height, and basal diameter), and Model 3 on four root morphology indicators (root length, root surface area, average root diameter, and root volume). PLSR reduces the dimensionality of the original variables, extracting key feature components, which are then used as new variables to build the regression model. The predictive variance of model for the dependent variable (Q^2^) was calculated using cross-validation. The root mean square error of estimation (RMSEE) was used to evaluate the accuracy of the model predictions. To prevent overfitting of the PLSR model, an appropriate number of PLSR components was determined by an optimal balance between R^2^ (model correlation coefficient, the explained variance of model for the dependent variables) and Q^2^. Variable importance for projection (VIP) is used to assess the degree of influence of the independent variables on the dependent variable. Phenolic compounds with a VIP > 1 and a standard regression coefficient< 0 are considered allelochemicals that inhibit the germination and seedling growth of *B. planiculmis* in reed aqueous extract (Zhang et al., 2020; Niazipoor et al., 2024).

### 2.6 Data analysis

Analysis of variance (ANOVA) was performed on germination, growth, and root morphology indicators of *B. planiculmis* using SPSS (version 25.0 (SPSS Inc., Chicago, USA). Before conducting the ANOVA, the normality of the data was assessed using the Shapiro-Wilk test, and the homogeneity of variance was tested using Levene’s test. The least significant difference test was performed to determine significant differences among treatments (*P* < 0.05). The Response Index (RI) was used to quantify the impact of a specific reed aqueous extract treatment on a particular indicator of *B. planiculmis*, where a positive value indicates promotion, a negative value indicates inhibition and the absolute value represents the intensity of the effect. The calculation formula is as follows (Bruce Williamson and Richardson, 1988):


(5) RI=1−CT  (when T ≥ C) or  RI=TC−1  (when T < C)

Where C represents the control value and T represents the treatment value. The synergistic allelopathic effect index (SE) is the arithmetic mean of the RI values for different measured indicators of *B. planiculmis* under the same reed aqueous extract treatment, thus quantifying the allelopathic activity of different extracts (Wang et al., 2024). The sensitivity index (SI) is the arithmetic mean of the RI values of the same indicator of *B. planiculmis* under different reed aqueous extract treatments; thus, it evaluates the sensitivity of the indicators to the treatment (Wang et al., 2024).

Principal component analysis (PCA) and partial least squares discriminant analysis (PLS-DA) were employed to evaluate the differences in the relative content of phenolic allelochemicals across different reed extracts. Phenolic allelochemicals with VIP > 1, a *P*-value from the t-test< 0.05, and fold change (FC) > 1.5, or FC< 0.67 were considered differential allelochemicals (Li et al., 2022). PLSR, PCA, and PLS-DA analyses were performed using the SIMCA17.0 software (Umetrics AB, Sweden).

## 3 Result

### 3.1 Effect of reed aqueous extracts on *Bolboschoenus planiculmis*

#### 3.1.1 Effect of reed aqueous extracts on the germination of *Bolboschoenus planiculmis*

**Figure 1:**
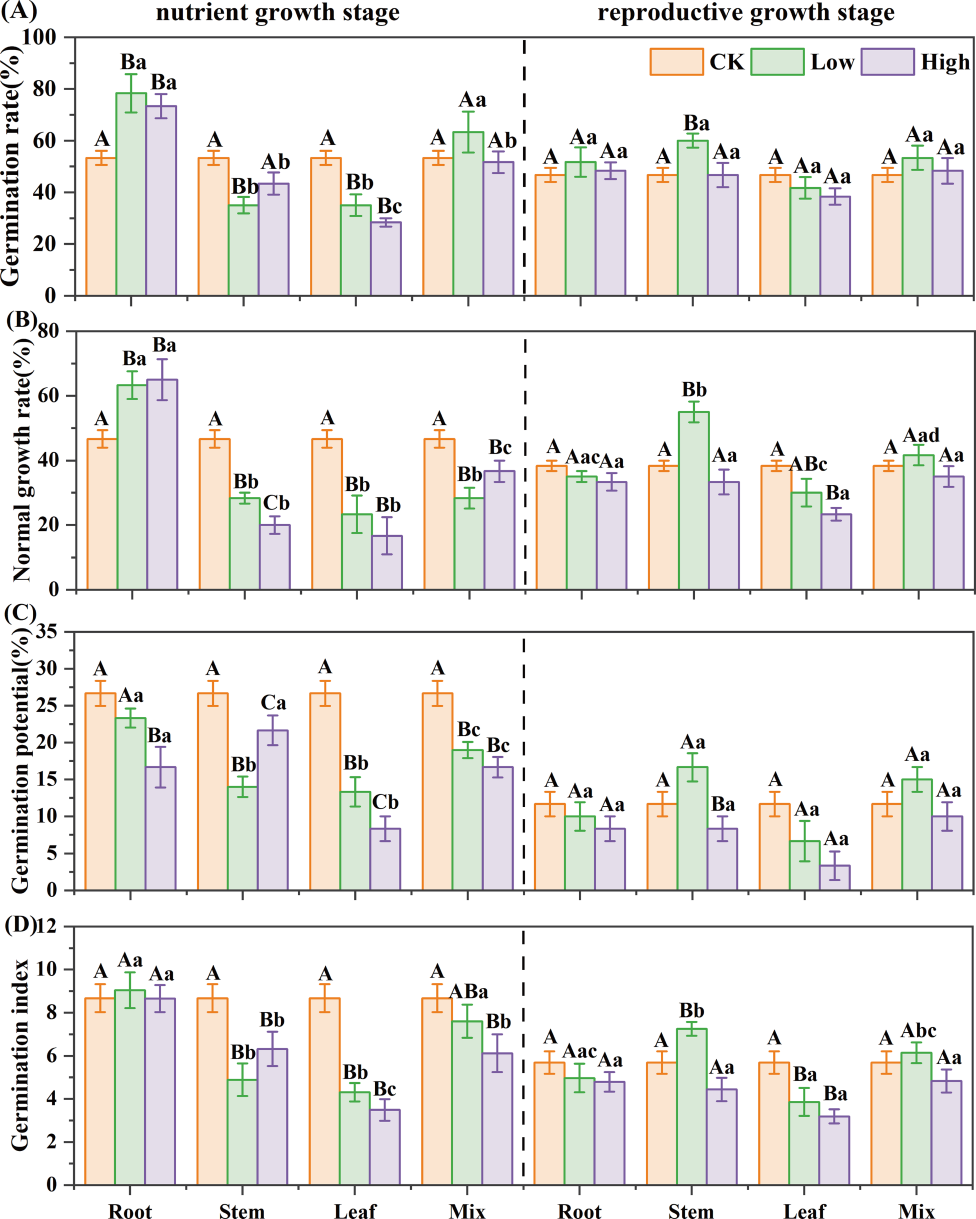
Effect of reed aqueous extracts on the germination of *Bolboschoenus planiculmis*. **(A)** germination rate, **(B)** normal growth rate, **(C)** germination potential, and **(D)** germination index. CK, Low, and High represent the control, low (7%), and high (14%) concentration aqueous extract, respectively. Different capital letters indicate significant differences (*P*< 0.05) between different concentrations of the same extract, while different lowercase letters indicate significant differences (*P*< 0.05) between different extracts at the same concentration.

Equations 1-4 were used to calculate the GR, NR, GP, and GI, respectively. For reed in the nutrient growth stage, stem, leaf, and mixed extracts inhibited the *B. planiculmis* germination, while root extract promoted it (**Figure 1**). The mixed extract reduced NR and GP by 21%–39%; stem extract reduced NR, GP, and GI by 9%–57%; leaf extract reduced all four germination indicators by 34%-69%; but root extract increased GR and NR by 36%–47% (*P*< 0.05). Among all treatments, the high-concentration leaf extract caused the greatest reduction in GR, NR, GP, and GI, with decreases of 47%, 64%, 69%, and 60% compared to the control, respectively. For reed in the reproductive growth stage, leaf and root extracts showed inhibitory effects on *B. planiculmis* germination, while stem and mixed extracts had a low-promoting, high-inhibitory effect (**Figure 1**). The leaf extract reduced GR, NR, GP, and GI by 11%–71%, and the root extract reduced NR, GP, and GI by 9%–29%. Among all treatments, the high-concentration leaf extract caused the greatest reduction in GR, NR, GP, and GI, with decreases of 18%, 39%, 71%, and 44% compared to the control, respectively. The interaction between reed growth stage and organ significantly affected the GR, NR, and GI of *B. planiculmis* (**Supplementary Table S2**, *P*< 0.001).

Based on Equation (5), RI was computed, and SE was subsequently determined. For reed in the nutrient growth stage, the SE of different extracts on germination was ranked as “LH< LL< SL< SH< MH< ML< RH< RL”. In the reproductive growth stage, it was “LH< LL< SH< RH< MH< RL< ML< SL” (**Table 2**). The leaf extract had the strongest inhibitory effect in both two growth stages. The root extract exhibited a promoting effect in the nutrient growth stage and an inhibitory effect in the reproductive growth stage.

**Table 2:** Allelopathic effect evaluation of reed aqueous extracts on germination of *Bolboschoenus planiculmis*.

Reed aqueous extracts		RI				SE
		GR	NR	GP	GI	
nutrient growth stage	RL	0.32	0.26	-0.13	0.04	0.12
	RH	0.27	0.28	-0.38	0.00	0.04
	SL	-0.34	-0.39	-0.44	-0.44	-0.40
	SH	-0.19	-0.57	-0.19	-0.27	-0.30
	LL	-0.34	-0.50	-0.50	-0.50	-0.46
	LH	-0.47	-0.64	-0.69	-0.60	-0.60
	ML	0.16	-0.39	-0.25	-0.12	-0.15
	MH	-0.03	-0.21	-0.38	-0.29	-0.23
	**SI**	-0.08	-0.27	-0.37	-0.27	-
reproductive growth stage	RL	0.10	-0.09	-0.14	-0.13	-0.07
	RH	0.03	-0.13	-0.29	-0.16	-0.14
	SL	0.22	0.30	0.30	0.21	0.26
	SH	0.00	-0.13	-0.29	-0.22	-0.16
	LL	-0.11	-0.22	-0.43	-0.32	-0.27
	LH	-0.18	-0.39	-0.71	-0.44	-0.43
	ML	0.12	0.09	0.22	0.07	0.13
	MH	0.03	-0.09	-0.14	-0.15	-0.09
	**SI**	0.03	-0.08	-0.18	-0.14	-

RL/RH, SL/SH, LL/LH, and ML/MH represent the low (L) and high (H) concentrations of aqueous extracts from the reed root (R), stem (S), leaf (L), and mixture (M), respectively. GR, germination rate; NR, normal growth rate; GP, germination potential; GI, germination index; RI, response index; SI, sensitivity index; SE, synergistic allelopathic effect index.

#### 3.1.2 Effect of reed aqueous extracts on the growth of *Bolboschoenus planiculmis* seedlings

##### 3.1.2.1 Effect of reed aqueous extracts on the growth morphology of *Bolboschoenus planiculmis* seedlings

**Figure 2:**
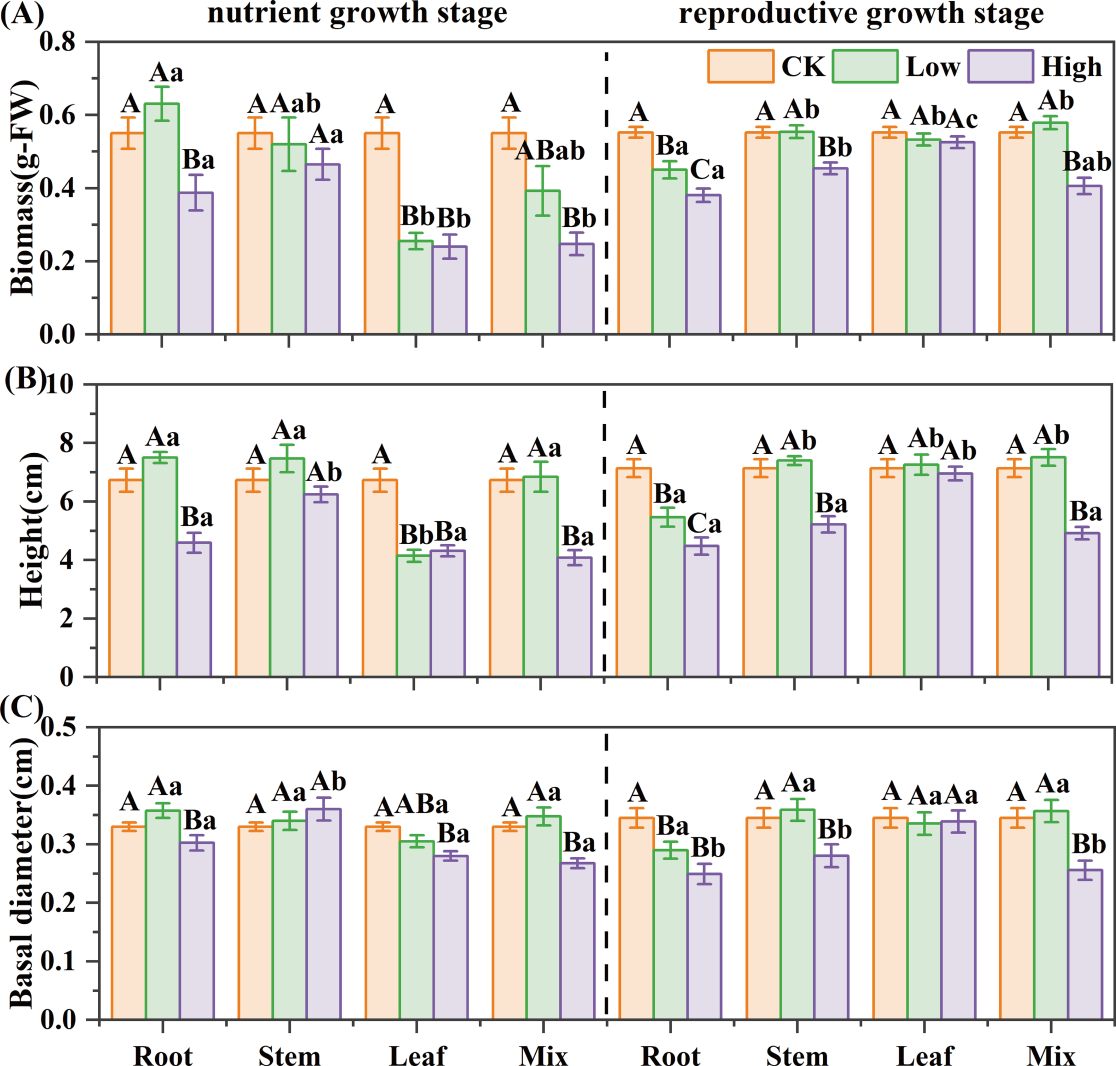
Effect of reed aqueous extracts on the growth morphology of *Bolboschoenus planiculmis* seedling. **(A)** biomass, **(B)** height, and **(C)** basal diameter. CK, Low, and High represent the control, low (7%), and high (14%) concentration aqueous extract, respectively. Different capital letters indicate significant differences (*P*< 0.05) between different concentrations of the same extract, while different lowercase letters indicate significant differences (*P*< 0.05) between different extracts at the same concentration.

For reed in the nutrient growth stage, the leaf extract strongly inhibited seedling growth of *B. planiculmis*, the mixed extract inhibited growth only at high concentrations, and the root extract showed a low-promoting, high-inhibitory effect (**Figure 2**). The high-concentration root extract reduced biomass, height, and basal diameter by 30%, 32%, and 8%, respectively; mixed extract by 55%, 39%, and 19%. The leaf extract significantly reduced biomass and height by 54% and 38% even at low concentrations (*P*< 0.05). For reed in the reproductive growth stage, the root extract inhibited the seedling growth of *B. planiculmis*, the stem and mixed extracts were inhibitory only at high concentrations (**Figure 2**). The high-concentration stem extract reduced biomass, height, and basal diameter by 18%, 27%, and 19%, respectively; mixed extract by 26%, 31%, and 26%; and root extract by 31%, 37%, and 28% (*P*< 0.05). The growth morphology of *B. planiculmis* seedlings was significantly influenced by the interaction among growth stage, organ, and concentration (**Supplementary Table S3**). The interactions between growth stage and organ, as well as between organ and concentration, significantly affected the biomass, height, and basal diameter of *B. planiculmis* (*P*< 0.05). The three-way interaction of these factors had a significant effect on seedling height and basal diameter (*P*< 0.05).

##### 3.1.2.2 Effect of reed aqueous extracts on the root morphology of *Bolboschoenus planiculmis* seedlings

**Figure 3:**
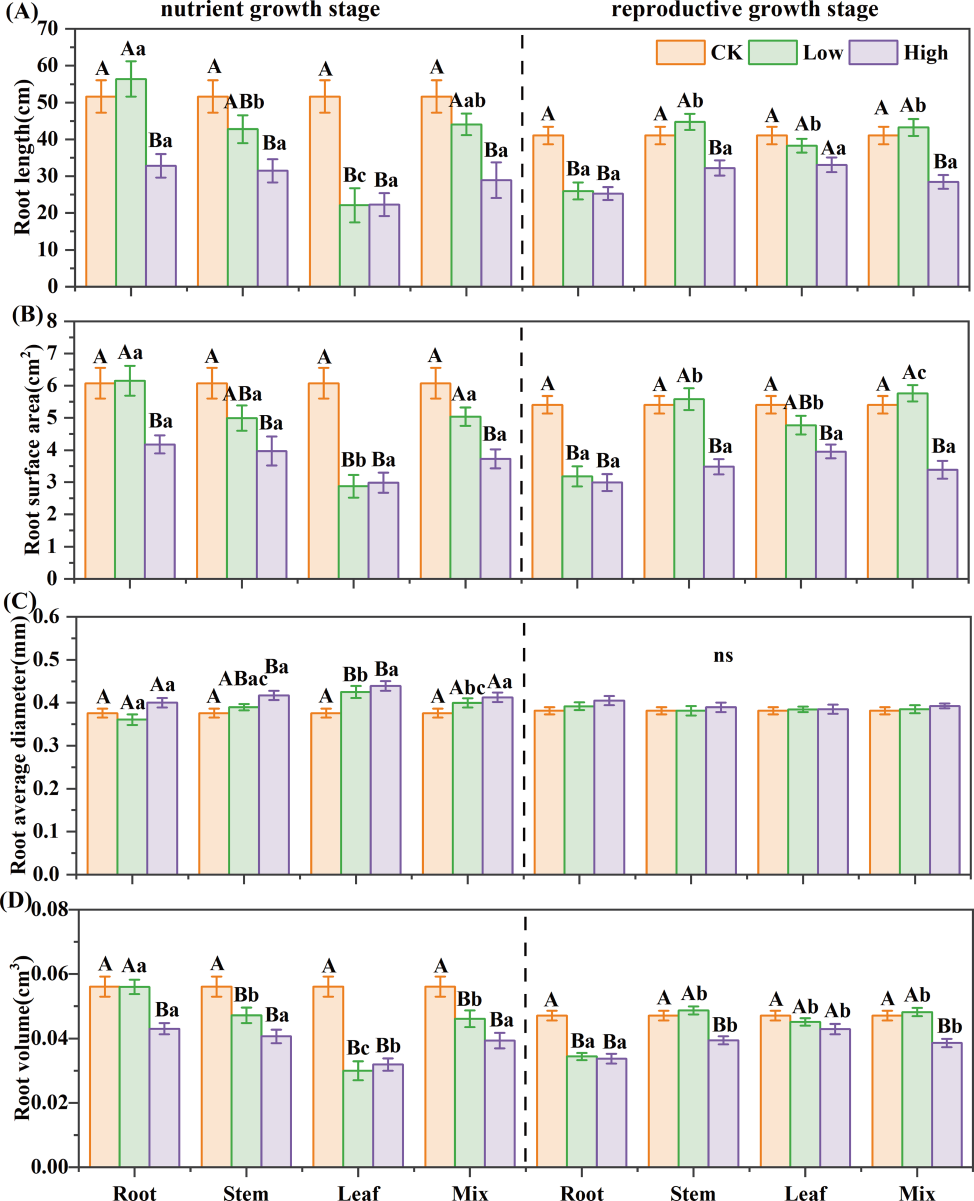
Effect of reed aqueous extracts on the root morphology of *Bolboschoenus planiculmis* seedling. **(A)** root length, **(B)** root surface area, **(C)** average root diameter, and **(D)** root volume. CK, Low, and High represent the control, low (7%), and high (14%) concentration aqueous extract, respectively. Different capital letters indicate significant differences (*P*< 0.05) between different concentrations of the same extract, while different lowercase letters indicate significant differences (*P*< 0.05) between different extracts at the same concentration.

For reed in the nutrient growth stage, stem, leaf, and mixed extracts inhibited root growth of *B. planiculmis*, the root extract was inhibitory only at high concentrations (**Figure 3**). The high-concentration root extract reduced root length, surface area, and volume by 36%, 31%, and 23%, respectively; stem extract by 39%, 35%, and 28%; and mixed extract by 44%, 39%, and 30%. The leaf extract exhibited strong inhibition even at low concentrations, reducing them by 57%, 53%, and 47% (*P*< 0.05). For reed in the reproductive growth stage, the leaf extract showed mild inhibition on root growth of *B. planiculmis*; stem and mixed extracts exhibited a low-promoting, high-inhibitory effect; and the root extract had a strong inhibitory effect even at low concentrations (**Figure 3**). The high-concentration root extract caused the greatest reduction in root length, root surface area, and root volume among all treatments, with decreases of 38%, 45%, and 28% compared to the control, respectively (*P*< 0.05). The root morphology of *B. planiculmis* seedlings was significantly influenced by the interaction among growth stage, organ, and concentration (**Supplementary Table S4**). The interactions between growth stage and organ, as well as between organ and concentration, significantly affected the root length, surface area, and volume of *B. planiculmis* (*P*< 0.05). The three-way interaction of these factors had a significant effect on root length and surface area (*P*< 0.05).

For reed in the nutrient growth stage, the SE of different extracts on seedling growth was ranked as “ LH = LL< MH< RH< SH< ML< SL< RL”. In the reproductive growth stage, it was “RH< MH< RL< SH< LH< LL< ML< SL” (**Table 3**). The leaf extract had the strongest inhibitory effect in the nutrient growth stage, while the root extract was most inhibitory in the reproductive growth stage.

**Table 3:** Allelopathic effect evaluation of reed aqueous extracts on the growth of *Bolboschoenus planiculmis* seedlings.

Reed aqueous extracts		RI							SE
		Biomass	Height	Basal diameter	Root length	Root surface area	Average root diameter	Root volume	
nutrient growth stage	RL	0.13	0.10	0.07	0.08	0.01	-0.04	0.00	0.05
	RH	-0.30	-0.32	-0.08	-0.36	-0.31	0.06	-0.23	-0.22
	SL	-0.06	0.10	0.04	-0.17	-0.18	0.04	-0.16	-0.06
	SH	-0.15	-0.07	0.08	-0.39	-0.35	0.10	-0.28	-0.15
	LL	-0.54	-0.38	-0.07	-0.57	-0.53	0.12	-0.47	-0.35
	LH	-0.56	-0.36	-0.15	-0.57	-0.51	0.14	-0.43	-0.35
	ML	-0.29	0.02	0.05	-0.15	-0.17	0.06	-0.18	-0.09
	MH	-0.55	-0.39	-0.19	-0.44	-0.39	0.09	-0.30	-0.31
	**SI**	-0.29	-0.16	-0.03	-0.32	-0.30	0.07	-0.26	–
reproductive growth stage	RL	-0.18	-0.23	-0.16	-0.37	-0.41	0.03	-0.27	-0.23
	RH	-0.31	-0.37	-0.28	-0.38	-0.45	0.06	-0.29	-0.29
	SL	0.00	0.03	0.04	0.08	0.03	0.00	0.03	0.03
	SH	-0.18	-0.27	-0.19	-0.22	-0.36	0.02	-0.16	-0.19
	LL	-0.04	0.02	-0.03	-0.07	-0.12	0.01	-0.04	-0.04
	LH	-0.05	-0.03	-0.02	-0.19	-0.27	0.01	-0.09	-0.09
	ML	0.05	0.05	0.03	0.05	0.06	0.01	0.02	0.04
	MH	-0.26	-0.31	-0.26	-0.31	-0.37	0.03	-0.18	-0.24
	**SI**	-0.12	-0.14	-0.11	-0.18	-0.24	0.02	-0.12	–

RL/RH, SL/SH, LL/LH, and ML/MH represent the low (L) and high (H) concentrations of aqueous extracts from the reed root (R), stem (S), leaf (L), and mixture (M), respectively. RI, response index; SI, sensitivity index; SE, synergistic allelopathic effect index.

### 3.2 Phenolic compounds in reed aqueous extracts

A total of 91 phenolic compounds were identified in reed aqueous extracts using untargeted metabolomics with HPLC-MS/MS, including 71 phenolic acids (**Supplementary Table S5**), 16 flavonoids (**Supplementary Table S6**), and four coumarins (**Supplementary Table S7**).

### 3.3 Phenolic allelochemicals in reed aqueous extracts

#### 3.3.1 Screening of phenolic allelochemicals

**Figure 4:**
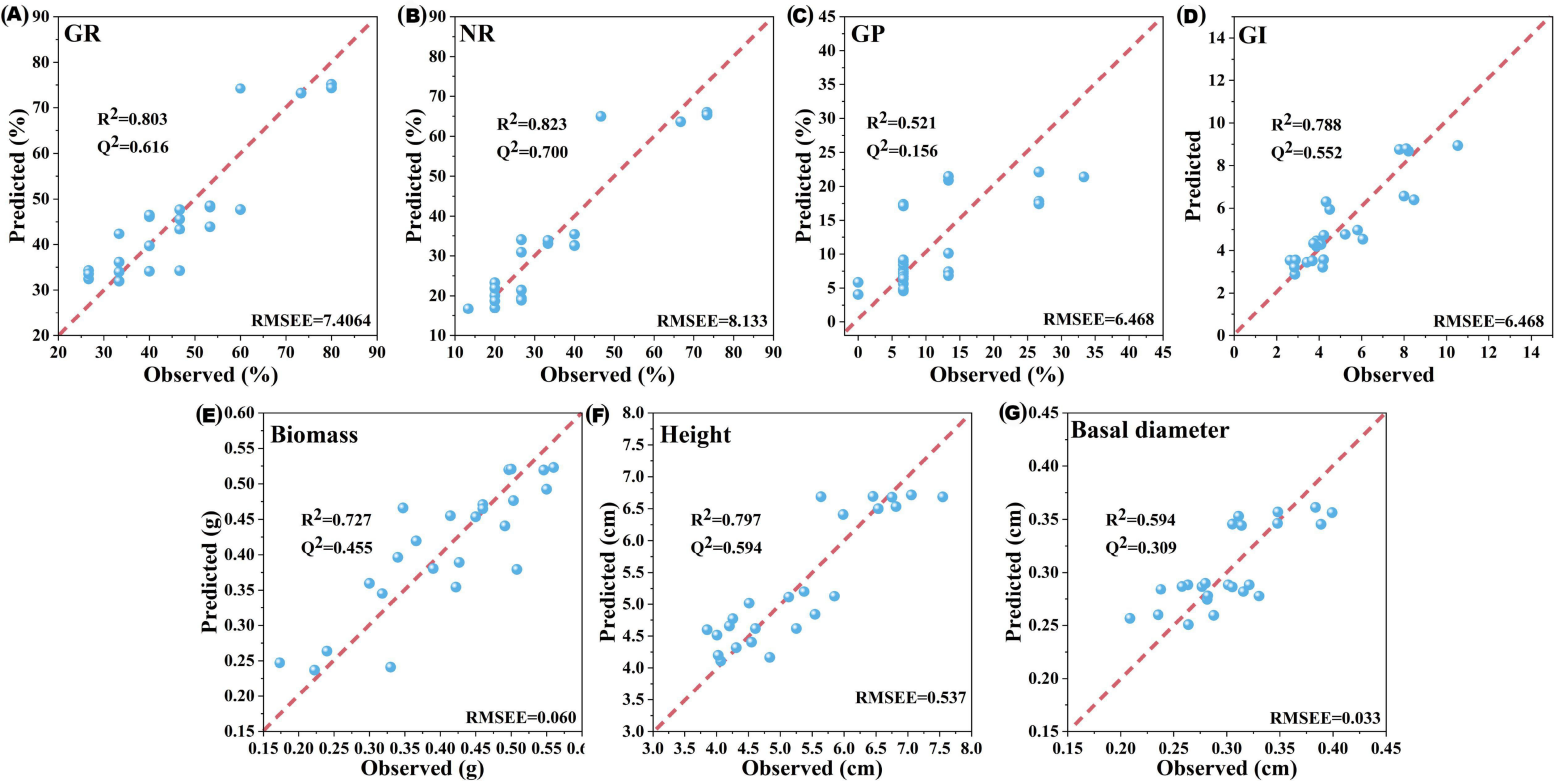
Comparison between model-predicted values and observed values of germination and seedling growth indicators for *Bolboschoenus planiculmis*. *R* ^2^ represents the variance explained by the model for each indicator. *Q* ^2^ represents the predictive variance of the model for each indicator. RMSEE was used to assess the prediction accuracy of the model for each indicator. **(A)** GR, germination rate; **(B)** NR, normal growth rate; **(C)** GP, germination potential; **(D)** GI, germination index; **(E)** Biomass; **(F)** Height; **(G)** Basal diameter.

The maximum Q^2^ values for M1 and M2 were obtained with three components, explaining 73.4% and 70.6% of the variance in the dependent variables, respectively (**Table 4**). M3 was excluded due to its poor explanatory and predictive performance. M1 and M2 exhibited strong predictive capabilities for the germination and seedling growth indicators of *B. planiculmis*, with minimal differences between predicted and observed values (**Figure 4**).

**Table 4:** Parameters of the PLSR models.

Model	Dependent variable	Component	R_X_^2^	R_X_^2^(cum)	R_Y_^2^	R_Y_^2^(cum)	Q^2^	Q^2^(cum)
M1	GR/NR/GP/GI	1	0.245	0.245	0.508	0.508	0.402	0.402
		2	0.277	0.522	0.124	0.632	0.116	0.471
		3	0.127	0.649	0.101	0.734	0.084	0.516
M2	Biomass/Height/Basal diameter	1	0.304	0.304	0.303	0.303	0.192	0.192
		2	0.159	0.463	0.253	0.556	0.187	0.343
		3	0.120	0.583	0.150	0.706	0.181	0.461
M3	Root length/Root surface area/Average root diameter/Root volume	1	0.177	0.177	0.359	0.359	0.033	0.033

R_X_^2^(cum) represents the cumulative explained variance of the component for the independent variable, R_Y_^2^(cum) represents the cumulative explained variance of the component for the dependent variable, and Q^2^(cum) represents the cumulative predictive variance of the component for the dependent variable.

**Figure 5:**
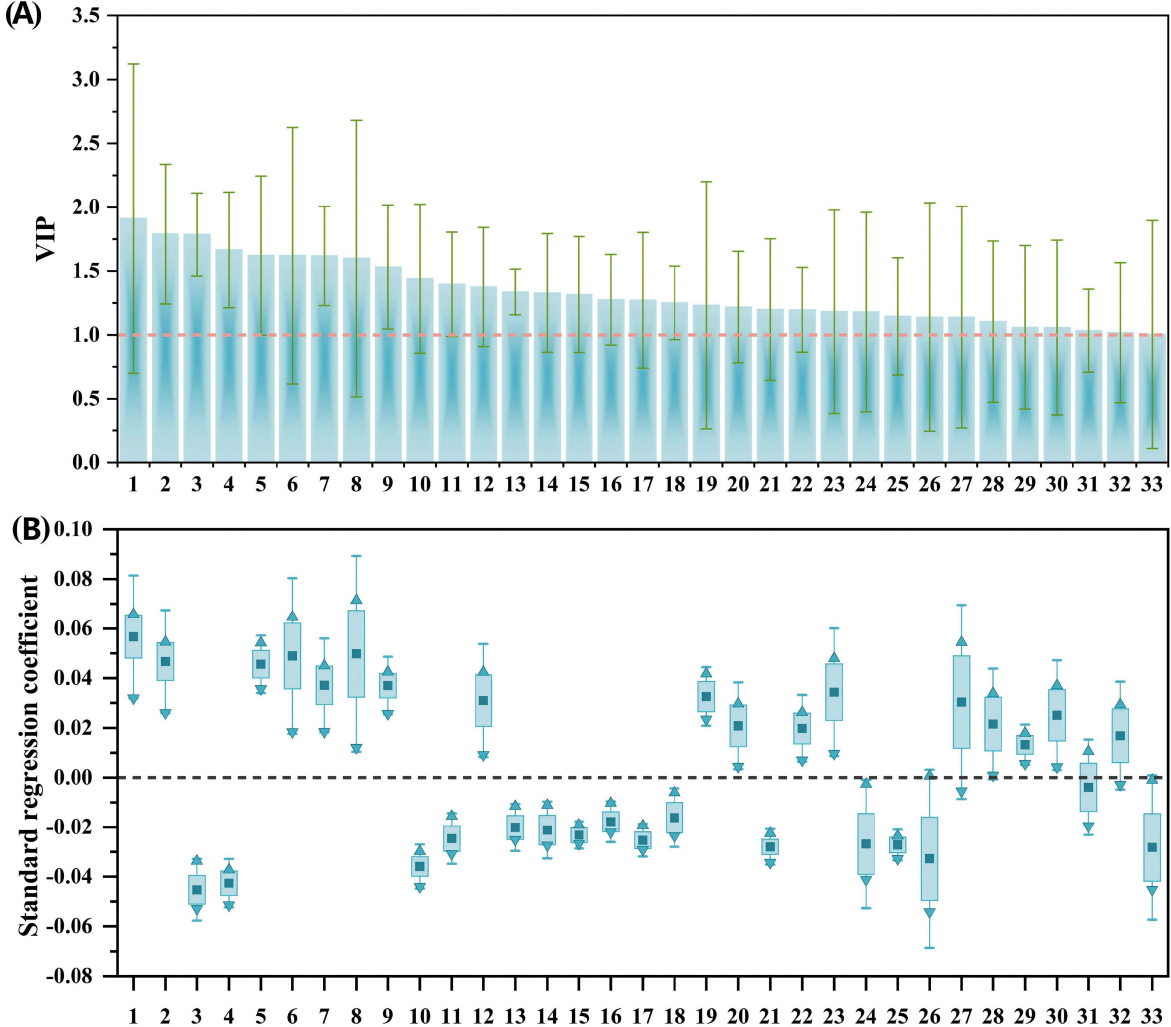
VIP values of phenolic compounds and their standard regression coefficients for the germination indicators of *Bolboschoenus planiculmis*. **(A)** VIP values; **(B)** Standard regression coefficients. Phenolic compounds (independent variables) are denoted by numbers on the x-axis. Only those with VIP values greater than 1 are included, along with their standardized regression coefficients for germination indicators. In panel **(B)**, dark squares, upward triangles, and downward triangles represent the mean, maximum, and minimum values of the standardized regression coefficients, respectively.

**Figure 6:**
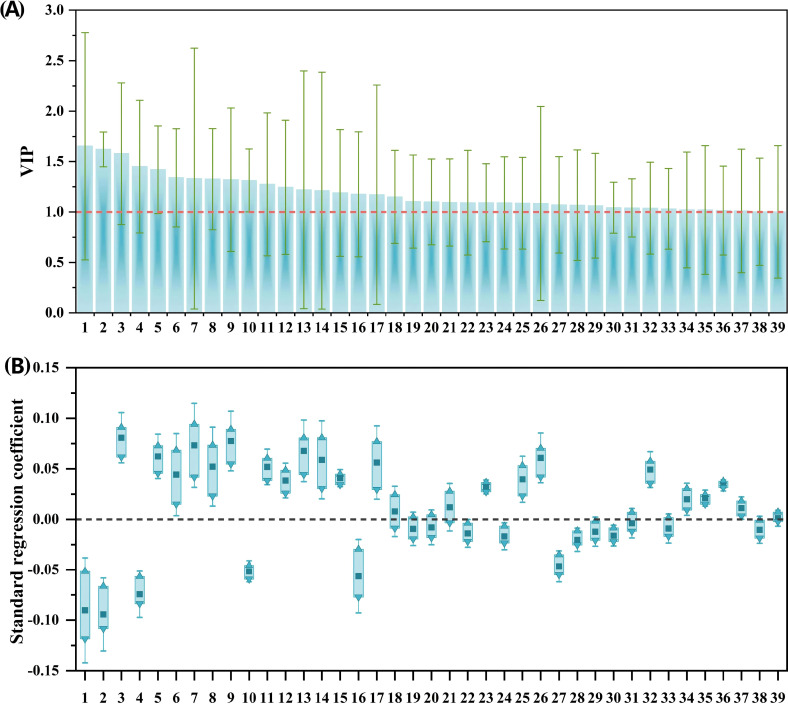
VIP values of phenolic compounds and their standard regression coefficients for the seedling growth indicators of *Bolboschoenus planiculmis*. **(A)** VIP values; **(B)** Standard regression coefficients. Phenolic compounds (independent variables) are denoted by numbers on the x-axis. Only those with VIP values greater than 1 are included, along with their standardized regression coefficients for seedling growth indicators. In panel **(B)**, dark squares, upward triangles, and downward triangles represent the mean, maximum, and minimum values of the standardized regression coefficients, respectively.

Fourteen potential phenolic allelochemicals that inhibit *B. planiculmis* germination were screened in reed aqueous extracts through M1 (**Figure 5**), and twelve allelochemicals that inhibit *B. planiculmis* seedling growth were screened through M2 (**Figure 6**). In total, 24 potential phenolic allelochemicals were identified, including 13 phenolic acids, nine flavonoids, and two coumarins (**Table 5**). 4-Hydroxypropranolol and rutin exhibited inhibitory effects on both germination and seedling growth of *B. planiculmis*.

**Table 5:** Potential phenolic allelochemicals in reed aqueous extracts.

Class	Number	Compound name	Inhibited process
Phenolic acid	1	4-Nitrophenol	germination
	2	3-Coumaric acid	germination
	3	6-Gingerol	germination
	4	Isorhapontigenin	germination
	5	4-Hexylresorcinol	germination
	6	Homogentisate	seedling growth
	7	Eugenol	seedling growth
	8	Vanillic acid	seedling growth
	9	Curcumin	seedling growth
	10	Ethyl ferulate	seedling growth
	11	Propylparaben	seedling growth
	12	Methyl 4-hydroxycinnamate	seedling growth
	13	4-Hydroxypropranolol	germination and seedling growth
Flavonoid	1	4’-O-Glucosylvitexin	germination
	2	Isorhamnetin	germination
	3	Orientin	germination
	4	Diosmetin	germination
	5	Quercetin-3β-D-glucoside	germination
	6	Quercetin	germination
	7	Quercetin-3-O-beta-glucopyranosyl-6’-acetate	germination
	8	Naringin	seedling growth
	9	Rutin	germination and seedling growth
Coumarin	1	Esculetin	seedling growth
	2	Isofraxidin	seedling growth

#### 3.3.2 Multivariate analysis of phenolic allelochemicals

**Figure 7:**
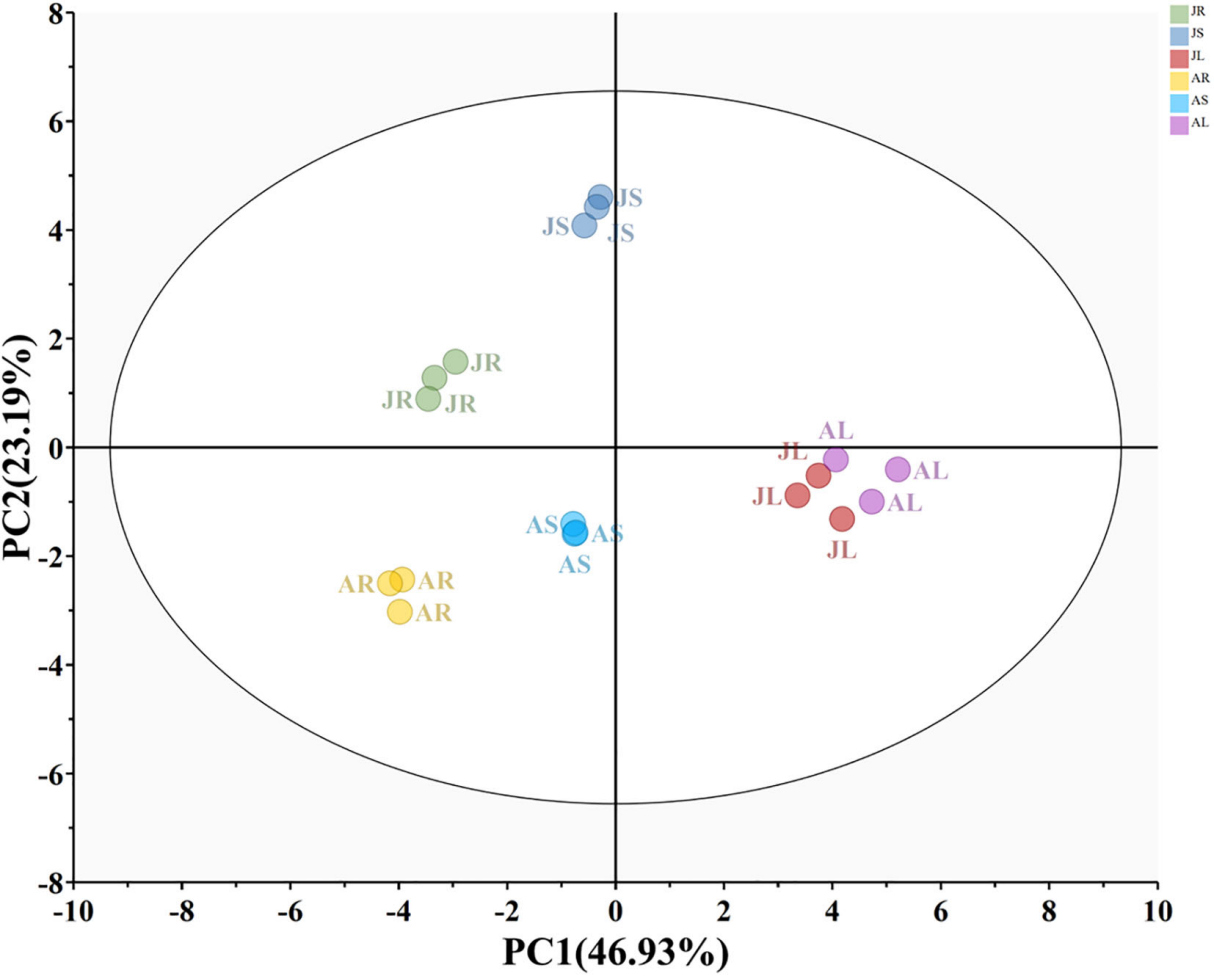
PCA of phenolic allelochemicals in reed aqueous extracts. JR, JS, and JL represent the root (R), stem (S), and leaf (L) extracts from the nutrient growth stage, respectively. AR, AS, and AL represent the root (R), stem (S), and leaf (L) extracts from the reproductive growth stage, respectively.

The PCA score plot revealed differences in the relative content of phenolic allelochemicals across different reed aqueous extracts (**Figure 7**). The first and second principal components distinguished the extracts based on organ type and growth stage, respectively. The content of phenolic allelochemicals in the leaf extract was significantly different from that in the stem and root extracts, with minimal variation between the two growth stages.

The leaf extract of reed during the nutrient growth stage contained significantly higher levels of 11 differential allelochemicals (VIP > 1, *P*< 0.05, and FC > 1.5 or FC< 0.67) compared to the stem extract from the same stage, with an average fold change of 46.47. Additionally, the contents of 12 differential allelochemicals in the leaf extract were significantly higher than those in the root extract during the nutrient growth stage, with an average fold change of 24.24 (**Table 6**).

**Table 6:** Differential allelochemicals among extracts from different organs of reed during the nutrient growth stage.

Class	Compound name	JL vs JR			JL vs JS		
		P	FC	VIP	P	FC	VIP
Phenolic acid	4-Nitrophenol	<0.001	2.97	1.07	<0.05	1.93	1.07
	6-Gingerol	<0.001	7.75	1.07	<0.001	3.76	1.07
	4-Hydroxypropranolol	<0.01	81.76	1.06	<0.01	14.78	1.06
	Isorhapontigenin	<0.001	16.16	1.07	<0.001	9.44	1.07
	3-Coumaric acid	<0.001	2.53	1.05	0.73	0.98	1.05
	Vanillic acid	<0.001	0.39	1.06	0.31	0.82	1.06
	Curcumin	<0.01	0.19	1.06	0.45	0.90	1.06
	Ethyl ferulate	<0.001	0.42	1.07	0.15	1.99	1.07
	Propylparaben	<0.01	0.52	1.00	0.87	0.91	1.00
	Methyl 4-hydroxycinnamate	<0.05	0.54	0.87	0.17	2.60	0.87
	4-Hexylresorcinol	0.11	11.12	0.87	0.11	23.84	0.87
	Homogentisate	0.24	1.25	0.60	<0.01	2.91	0.60
	Eugenol	0.21	0.52	0.72	<0.01	1.78	0.72
Flavonoid	4’-O-Glucosylvitexin	<0.001	44.04	1.07	<0.001	40.13	1.07
	Orientin	<0.001	11.63	1.07	<0.001	143.97	1.07
	Rutin	<0.01	46.17	1.06	<0.01	50.42	1.06
	Diosmetin	<0.01	28.50	1.06	<0.01	9.26	1.06
	Quercetin-3β-D-glucoside	<0.001	15.53	1.05	<0.01	106.46	1.05
	Quercetin	<0.05	22.29	1.04	<0.01	78.97	1.04
	Quercetin-3-O-beta-glucopyranosyl-6’-acetate	<0.001	11.53	1.05	<0.01	52.01	1.05
	Naringin	0.06	1.69	0.85	<0.001	9.76	0.85
	Isorhamnetin	<0.01	1.35	1.00	0.13	0.61	1.00
Coumarin	Isofraxidin	<0.001	0.16	1.06	<0.05	1.30	1.06
	Esculetin	<0.05	0.62	0.95	<0.05	3.56	0.95

JR, JS, and JL refer to the high-concentration root (R), stem (S), and leaf (L) extracts of reed during the nutrient growth stage, respectively. *P* represents the statistical significance from univariate analysis (t-test). FC indicates the fold change in the relative content of phenolic allelochemicals. VIP represents the variable importance for projection based on PLS-DA. Compounds with *P*< 0.05, VIP > 1, and FC > 1.5 or FC< 0.67 are considered differential allelochemicals between the two aqueous extracts.

## 4 Discussion

The results confirm the allelopathic activity of reed against *B. planiculmis*, as reed aqueous extracts significantly inhibited its germination and seedling growth. Previous studies also found such allelopathic effects of reed on other plants (Uddin and Robinson, 2017a; Uddin et al., 2017; Gao et al., 2022; Wang C. H. et al., 2022). For example, reed aqueous extracts have been reported to reduce the germination rate of *A. fatua* (Ahmad Afridi and Khan, 2015), decrease the root number in *Mucuna pruriens* (Uddin et al., 2017), shorten the radicle length of *S. salsa* (Gao et al., 2022), and suppress both plant height and biomass in *Rumex crispus* (Ahmad Afridi and Khan, 2015). In this study, reed aqueous extracts significantly reduced the germination rate and germination speed of *B. planiculmis* and strongly inhibited its root growth. Specifically, root length, surface area, and volume were reduced by up to 57%, 53%, and 43%, respectively, compared with the control. Unlike previous studies that used filter paper as the growth substrate in bioassays (Wang C. H. et al., 2022; Gao et al., 2022; Uddin and Robinson, 2017a), this study employed *in situ* soil collected from natural *B. planiculmis* populations. Under natural conditions, soil can modulate the allelopathic effects of reed through multiple mechanisms. On one hand, the physical adsorption and microbial degradation of allelochemicals by the soil can reduce their direct inhibitory impact on plants (Revillini et al., 2023; Kong et al., 2024; Yuan et al., 2024). On the other hand, allelopathic interactions may alter the structure and composition of the soil microbial community, thereby indirectly intensifying the inhibitory effects on plant growth (Ge et al., 2018; Zuo et al., 2022). Moreover, studies have shown that soil salinity and water availability can influence the allelopathic potential of plants by regulating the synthesis and release of allelochemicals (Nornasuha and Ismail, 2017; Han et al., 2024; Kostina-Bednarz et al., 2023; Shan et al., 2023). Reed typically inhabits wetland ecosystems characterized by frequent fluctuations in water levels and salinity. Therefore, in natural communities, the strength of reed allelopathic effects against *B. planiculmis* may fluctuate in response to habitat variations.

Uddin et al. (2014a) compared the allelopathic activity of aqueous extracts from different reed organs and found that the leaves exhibited the strongest inhibitory activity, followed by rhizomes, roots, stems, and inflorescences. In contrast, Gao et al. (2022) reported that the underground parts displayed greater inhibition activity than the aboveground parts. This study assessed the allelopathic activity of reed at two growth stages and revealed that its allelopathic activity is influenced by both the developmental stage and the plant organ. This may partly account for the inconsistencies reported in previous research. In this study, reed leaves from both growth stages exhibited notable allelopathic inhibitory activity. In particular, aqueous extracts of leaves collected during the nutrient stage significantly suppressed the seed germination and seedling growth of *B. planiculmis*, even at low concentrations. Similar findings have been reported in other allelopathic species such as *Juglans regia* (Đorđević et al., 2022), *Asclepias syriaca* (Gajić Umiljendić et al., 2025), and *Parthenium hysterophorus* (Bashar et al., 2022), whose leaf extracts also exhibit strong allelopathic inhibition activity and considerable potential for development as bioherbicides. As a perennial species with high biomass, reed produces large quantities of leaf litter annually, which accumulates in soil or aquatic environments (Packer et al., 2017). Under natural conditions, reed leaves are likely a major source of its allelopathic activity on *B. planiculmis*.

Previous studies have confirmed that phenolic acids are key active allelochemicals present in reed root exudates and decomposition products (Rudrappa et al., 2007; Nakai et al., 2006). This study demonstrates that phenolic acids are also important active constituents in reed aqueous extracts and reports several phenolic acids that had not been previously identified in this species. Among the 24 potential phenolic allelochemicals identified, 13 were classified as phenolic acids, 12 of which—excluding vanillic acid—are reported for the first time in reed (**Table 5**). Moreover, this study is the first to reveal that the allelopathic activity of reed originates not only from phenolic acids but also from flavonoids and coumarins. The biological activity of these potential allelochemicals has also been reported in other studies. Phytotoxicity studies demonstrated that rutin can inhibit root growth, chlorophyll content, and PSII photosynthetic efficiency of *A. thaliana* (Hussain and Reigosa, 2014, 2016). Notably, rutin has been proposed as a promising natural herbicide due to its allelopathic inhibition (Hussain and Reigosa, 2014; Fonseca et al., 2017). Xin et al. (2022) reported that quercetin significantly suppressed the growth of *L. sativa* seedlings at 50 μg/mL. Facenda et al. (2023) confirmed that esculetin at a concentration of 30 mg/L can significantly inhibit root growth in *L. sativa*. Hu et al. (2023) reported that isofraxidin exhibited notable activity against planktonic *Staphylococcus aureus*, with an IC_50_ value of 13.51 µg/mL. The strong bioherbicidal potential of esculetin and isofraxidin is attributed to the presence of a hydroxyl group at the C7 position in their molecular structures (Pan et al., 2015; Zhao et al., 2021). Therefore, in reed, the aforementioned compounds may individually affect germination and seedling growth of *B. planiculmis*. Furthermore, synergistic effects have also been observed among phenolic allelochemicals (Huang et al., 2020). α-Amylase plays a crucial role in breaking down starch to supply energy for plant growth. Research has demonstrated that quercetin and rutin can synergistically inhibit α-amylase activity, thereby suppressing seed germination (Oboh et al., 2015). Our study found that the concentrations of rutin and quercetin in reed leaves were significantly higher than those in the roots and stems (**Table 6**). The stronger allelopathic inhibition observed in the leaves may be attributed to the enhanced synergistic and additive interactions among the allelochemicals they contain.

The concentration of phenolic allelochemicals varies among different reed organs. Leaves are recognized as primary storage sites for phenolic secondary metabolites in plants (Pinhatti et al., 2010). Silva et al. (2007) proposed that photosynthetic activity promotes the accumulation of flavonoids and phenolic acids in leaves. Studies have reported high concentrations of phenolic compounds in the leaves of various species (Sultana and Anwar, 2008; Chen et al., 2019). Consistent with previous findings, this study demonstrated that the concentration of phenolic allelochemicals in reed leaves is much higher than that in roots and stems, which may account for the greater allelopathic inhibition activity of reed leaf extracts. Reed produces abundant biomass, with its leaves typically shedding onto the soil surface at the end of the growing season. In natural wetlands, these reeds often form thick layers of litter accumulating on the ground (Packer et al., 2017). Considering the potential allelopathic inhibitory effects of reed on co-occurring species, regular monitoring of reed biomass, along with periodic removal of litter deposits, are essential.

## 5 Conclusion

This study confirms that reed exerts significant allelopathic inhibitory effects on the germination and seedling growth of *B. planiculmis*. In natural wetland ecosystems, such allelopathy may pose a potential threat to the individual performance and population dynamics of *B. planiculmis*. A total of 24 phenolic compounds were identified in reed extracts, constituting the chemical basis of its allelopathic inhibitory activity. Among different reed organs, leaves were identified as the important source of allelopathic inhibitory activity, with the concentrations of phenolic allelochemicals substantially higher than those in roots and stems. Importantly, this study is the first to demonstrate that, in addition to phenolic acids, flavonoids and coumarins also serve as key allelochemicals in reed.

## Data Availability

The original contributions presented in the study are included in the article/**Supplementary Material**. Further inquiries can be directed to the corresponding authors.
