# Supplementary material for: Allelopathic activity of Phragmites australis against Bolboschoenus planiculmis and the involved active allelochemicals
Source: Front Plant Sci. 2025 Jun 23;16:1607628. doi: 10.3389/fpls.2025.1607628 (PMC12230101; doi:10.3389/fpls.2025.1607628)
Supplement: Supplementary file 1 [file Table1.docx]

***Supplementary Material***

**Supplementary Table S1.** The effect of reed aqueous extracts on the biomass of *Bolboschoenus planiculmis* seedlings (preliminary experiments)

| **Extracts** | **Concentration (m/v%)** | **Biomass (FW g)** |
| --- | --- | --- |
| Root | 0 | 0.64 |
|  | 3.5 | 0.67 |
|  | 7 | 0.735* |
|  | 14 | 0.57* |
| Stem | 0 | 0.64 |
|  | 3.5 | 0.65 |
|  | 7 | 0.68 |
|  | 14 | 0.61 |
| Leaf | 0 | 0.64 |
|  | 3.5 | 0.60 |
|  | 7 | 0.58* |
|  | 14 | 0.55* |

Note: FW refers to fresh weight; “*” indicates significant differences (*P*<0.05) compared to the 0% concentration.

**Supplementary Table S2.** Three-way ANOVA of the effects of different factors on the germination of *Bolboschoenus planiculmis*

| **Source of variation** | **GR** | | **NR** | | **GP** | | **GI** | |
| --- | --- | --- | --- | --- | --- | --- | --- | --- |
|  | **F** | ***P*** | **F** | ***P*** | **F** | ***P*** | **F** | ***P*** |
| Growth stage | 1.13 | 0.30 | 0.11 | 0.75 | 14.57 | **<0.001** | 16.43 | **<0.001** |
| Organ | 24.05 | **<0.001** | 30.65 | **<0.001** | 3.83 | **<0.05** | 16.05 | **<0.001** |
| Concentration | 4.52 | **<0.05** | 7.41 | **<0.01** | 3.23 | 0.08 | 5.29 | **<0.05** |
| Growth stage×Organ | 14.43 | **<0.001** | 31.35 | **<0.001** | 0.45 | 0.72 | 7.55 | **<0.001** |
| Growth stage×  Concentration | 0.28 | 0.60 | 4.28 | **<0.05** | 0.45 | 0.50 | 1.91 | 0.17 |
| Organ×Concentration | 0.27 | 0.85 | 3.68 | **<0.05** | 0.20 | 0.89 | 0.46 | 0.71 |
| Growth stage×  Organ×Concentration | 1.90 | 0.14 | 0.93 | 0.43 | 1.40 | 0.26 | 2.67 | 0.06 |

Note: germination rate (GR); normal growth rate (NR); germination potential (GP); germination index (GI). Significant effects (*P*<0.05) are in bold.

**Supplementary Table S3.** Three-way ANOVA of the effects of different factors on the growth morphology of *Bolboschoenus planiculmis* seedlings

| **Source of variation** | **Biomass** | | **Height** | | **Basal diameter** | |
| --- | --- | --- | --- | --- | --- | --- |
|  | **F** | ***P*** | **F** | ***P*** | **F** | ***P*** |
| Growth stage | 25.53 | **<0.001** | 11.15 | **<0.01** | 2.42 | 0.13 |
| Organ | 7.61 | **<0.001** | 10.02 | **<0.001** | 3.78 | **<0.05** |
| Concentration | 30.03 | **<0.001** | 113.68 | **<0.001** | 30.49 | **<0.001** |
| Growth stage×Organ | 20.45 | **<0.001** | 34.50 | **<0.001** | 7.78 | **<0.001** |
| Growth stage×Concentration | 0.53 | 0.47 | 0.31 | 0.58 | 1.36 | 0.25 |
| Organ×Concentration | 3.75 | **<0.05** | 13.46 | **<0.001** | 4.77 | **<0.01** |
| Growth stage×  Organ×Concentration | 1.87 | 0.15 | 4.40 | **<0.01** | 3.37 | **<0.05** |

Note: Significant effects (*P*<0.05) are in bold.

**Supplementary Table S4.** Three-way ANOVA of the effects of different factors on the root morphology of *Bolboschoenus planiculmis* seedlings

| **Source of variation** | **Root length** | | **Root  surface area** | | **Average root diameter** | | **Root volume** | |
| --- | --- | --- | --- | --- | --- | --- | --- | --- |
|  | **F** | ***P*** | **F** | ***P*** | **F** | ***P*** | **F** | ***P*** |
| Growth stage | 0.59 | 0.45 | 0.40 | 0.53 | 10.11 | **<0.01** | 0.41 | 0.53 |
| Organ | 6.21 | **<0.01** | 6.25 | **<0.01** | 2.45 | 0.07 | 9.57 | **<0.001** |
| Concentration | 44.73 | **<0.001** | 57.17 | **<0.001** | 9.17 | **<0.01** | 38.38 | **<0.001** |
| Growth stage×Organ | 18.59 | **<0.001** | 20.77 | **<0.001** | 6.24 | **<0.01** | 26.06 | **<0.001** |
| Growth stage×  Concentration | 1.80 | 0.19 | 1.00 | 0.32 | 2.22 | 0.14 | 0.34 | 0.56 |
| Organ×Concentration | 3.06 | **<0.05** | 4.13 | **<0.05** | 0.61 | 0.61 | 4.54 | **<0.01** |
| Growth stage×  Organ×Concentration | 4.19 | **<0.05** | 4.84 | **<0.01** | 0.14 | 0.93 | 2.67 | 0.06 |

Note: Significant effects (*P*<0.05) are in bold.

**Supplementary Table S5.** Phenolic acids in reed aqueous extracts

| **Number** | **Compound** | **Formula** | **Molecular Weight** | **RT [min]** | **m/z** |
| --- | --- | --- | --- | --- | --- |
| 1 | 3,5-Dihydroxybenzoic acid | C_7_H_6_O_4_ | 154.03 | 5.43 | 153.02 |
| 2 | 3-Hydroxybenzoic acid | C_7_H_6_O_3_ | 138.03 | 5.26 | 137.02 |
| 3 | 2,4-Dihydroxybenzoic acid | C_7_H_6_O_4_ | 154.03 | 5.23 | 155.03 |
| 4 | 4-Hydroxybenzoic acid | C_7_H_6_O_3_ | 138.03 | 5.50 | 139.04 |
| 5 | Protocatechuic acid | C_7_H_6_O_4_ | 154.03 | 4.68 | 155.03 |
| 6 | 2,3-Dihydroxybenzoic acid | C_7_H_6_O_4_ | 154.03 | 4.90 | 153.02 |
| 7 | 2,6-Dihydroxybenzoic acid | C_7_H_6_O_4_ | 154.03 | 4.97 | 155.03 |
| 8 | Gallic acid | C_7_H_6_O_5_ | 170.02 | 2.90 | 169.01 |
| 9 | Butylparaben | C_11_H_14_O_3_ | 194.09 | 6.56 | 193.09 |
| 10 | Propylparaben | C_10_H_12_O_3_ | 180.08 | 6.50 | 179.07 |
| 11 | Ethylparaben | C_9_H_10_O_3_ | 166.06 | 4.20 | 167.07 |
| 12 | Methyl gallate | C_8_H_8_O_5_ | 184.04 | 5.06 | 183.03 |
| 13 | Syringic acid | C_9_H_10_O_5_ | 198.05 | 5.35 | 197.05 |
| 14 | Isovanillic acid | C_8_H_8_O_4_ | 168.04 | 4.90 | 169.05 |
| 15 | Vanillic acid | C_8_H_8_O_4_ | 168.04 | 5.15 | 167.03 |
| 16 | 2-Hydroxycinnamic acid | C_9_H_8_O_3_ | 164.05 | 5.28 | 163.04 |
| 17 | 3-Coumaric acid | C_9_H_8_O_3_ | 164.05 | 5.49 | 163.04 |
| 18 | 4-Coumaric acid | C_9_H_8_O_3_ | 164.05 | 2.35 | 165.05 |
| 19 | Caffeic acid | C_9_H_8_O_4_ | 180.04 | 5.29 | 179.04 |
| 20 | Ethyl ferulate | C_12_H_14_O_4_ | 222.09 | 5.06 | 221.08 |
| 21 | Ferulic acid | C_10_H_10_O_4_ | 194.06 | 5.60 | 193.05 |
| 22 | Isoferulic acid | C_10_H_10_O_4_ | 194.06 | 5.14 | 195.07 |
| 23 | Methyl 4-hydroxy-3-methoxycinnamate | C_11_H_12_O_4_ | 208.07 | 4.80 | 209.08 |
| 24 | Methyl 4-hydroxycinnamate | C_10_H_10_O_3_ | 178.06 | 3.89 | 179.07 |
| 25 | Methyl cinnamate | C_10_H_10_O_2_ | 162.07 | 4.73 | 163.08 |
| 26 | Sinapinic acid | C_11_H_12_O_5_ | 224.07 | 5.69 | 223.06 |
| 27 | trans-Cinnamic acid | C_9_H_8_O_2_ | 148.05 | 5.66 | 147.05 |
| 28 | 3-(4-Hydroxyph-enyl) propionic acid | C_9_H_10_O_3_ | 166.06 | 5.22 | 165.06 |
| 29 | 3,4-Dihydroxyp-henylpropionic acid | C_9_H_10_O_4_ | 182.06 | 5.51 | 181.05 |
| 30 | DL-4-Hydroxyp-henyllactic acid | C_9_H_10_O_4_ | 182.06 | 5.09 | 181.05 |
| 31 | Catechol | C_6_H_6_O_2_ | 110.04 | 5.21 | 109.03 |
| 32 | Resorcinol | C_6_H_6_O_2_ | 110.04 | 1.72 | 109.03 |
| 33 | Phloroglucinol | C_6_H_6_O_3_ | 126.03 | 4.70 | 125.02 |
| 34 | Pyrogallol | C_6_H_6_O_3_ | 126.03 | 2.92 | 125.02 |
| 35 | 4-Ethylphenol | C_8_H_10_O | 122.07 | 5.56 | 121.07 |
| 36 | 4-Hexylresorcinol | C_12_H_18_O_2_ | 194.13 | 5.51 | 193.12 |
| 37 | 4-Methylcatechol | C_7_H_8_O_2_ | 124.05 | 4.55 | 123.05 |
| 38 | 4-Methylphenol | C_7_H_8_O | 108.06 | 5.48 | 107.05 |
| 39 | 4-tert-butylphenol | C_10_H_14_O | 150.10 | 5.72 | 149.10 |
| 40 | 4-tert-Amylphenol | C_11_H_16_O | 164.12 | 6.43 | 163.11 |
| 41 | 4-Nitrocatechol | C_6_H_5_NO_4_ | 155.02 | 3.14 | 154.01 |
| 42 | 4-Nitrophenol | C_6_H_5_NO_3_ | 139.03 | 5.81 | 138.02 |
| 43 | Terbutaline | C_12_H_19_NO_3_ | 225.14 | 4.76 | 226.14 |
| 44 | 3',4'-Dihyd-roxyphenylacetone | C_9_H_10_O_3_ | 166.06 | 5.34 | 165.06 |
| 45 | Salicylic acid | C_7_H_6_O_3_ | 138.03 | 11.97 | 137.02 |
| 46 | 5-Sulfosalicylic acid | C_7_H_6_O_6_S | 217.99 | 4.70 | 216.98 |
| 47 | 5-Methoxysalicylic acid | C_8_H_8_O_4_ | 168.04 | 5.33 | 167.04 |
| 48 | Anacardic acid | C_22_H_36_O_3_ | 348.27 | 7.49 | 349.27 |
| 49 | O-Acetyl-5-aminosalicylic acid | C_9_H_9_NO_4_ | 195.05 | 2.64 | 196.06 |
| 50 | Mesalamine | C_7_H_7_NO_3_ | 153.04 | 2.37 | 152.04 |
| 51 | 2-Methoxy-4-vinylphenol | C_9_H_10_O_2_ | 150.07 | 4.88 | 149.06 |
| 52 | Coniferyl alcohol | C_10_H_12_O_3_ | 180.08 | 5.55 | 179.07 |
| 53 | Eugenol | C_10_H_12_O_2_ | 164.08 | 5.03 | 163.08 |
| 54 | Homovanillic acid | C_9_H_10_O_4_ | 182.06 | 4.83 | 181.05 |
| 55 | Vanillyl alcohol | C_8_H_10_O_3_ | 154.06 | 5.03 | 153.06 |
| 56 | 1-Naphthol | C_10_H_8_O | 144.06 | 4.80 | 143.05 |
| 57 | 4-Hydroxypropranolol | C_16_H_21_NO | 275.15 | 4.81 | 276.16 |
| 58 | Isorhapontigenin | C_15_H_14_O_4_ | 258.09 | 6.33 | 257.08 |
| 59 | Oxyresveratrol | C_14_H_12_O_4_ | 244.07 | 5.67 | 243.07 |
| 60 | Polydatin | C_20_H_22_O_8_ | 390.13 | 5.38 | 389.12 |
| 61 | Curcumin | C_21_H_20_O_6_ | 368.13 | 5.40 | 369.13 |
| 62 | 2-Hydroxyhippuric acid | C_9_H_9_NO_4_ | 195.05 | 5.96 | 194.05 |
| 63 | 4-Hydroxybenzylcyanide | C_8_H_7_NO | 133.05 | 4.76 | 178.05 |
| 64 | 4-Hydroxyphenylpyruvic acid | C_9_H_8_O_4_ | 180.04 | 3.87 | 179.03 |
| 65 | Vanillin | C_8_H_8_O_3_ | 152.05 | 3.12 | 151.04 |
| 66 | 4-Hydroxybenzylalcohol | C_7_H_8_O_2_ | 124.05 | 5.12 | 123.05 |
| 67 | Homogentisate | C_8_H_8_O_4_ | 168.04 | 1.89 | 169.05 |
| 68 | 6-Gingerol | C_17_H_26_O_4_ | 294.18 | 6.73 | 295.19 |
| 69 | 6-Shogaol | C_17_H_24_O_3_ | 276.17 | 6.44 | 277.18 |
| 70 | Capsaicin | C_18_H_27_NO_3_ | 305.20 | 5.23 | 306.21 |
| 71 | Sinapyl aldehyde | C_11_H_12_O_4_ | 208.07 | 3.03 | 209.08 |

Note: RT represents retention time; m/z is the mass-to-charge ratio.

**Supplementary Table S6.** Flavonoids in reed aqueous extracts

| **Number** | **Compound** | **Formula** | **Molecular Weight** | **RT [min]** | **m/z** |
| --- | --- | --- | --- | --- | --- |
| 1 | Apigenin | C_15_H_10_O_5_ | 270.05 | 6.13 | 269.05 |
| 2 | Biochanin A | C_16_H_12_O_5_ | 284.07 | 6.92 | 285.08 |
| 3 | Diosmetin | C_16_H_12_O_6_ | 300.06 | 6.15 | 301.07 |
| 4 | Luteolin | C_15_H_10_O_6_ | 286.05 | 5.93 | 285.04 |
| 5 | Glycitein | C_16_H_12_O_5_ | 284.07 | 6.91 | 283.06 |
| 6 | Hesperetin | C_16_H_14_O_6_ | 302.08 | 6.11 | 301.07 |
| 7 | Quercetin | C_15_H_10_O_7_ | 302.04 | 5.84 | 301.04 |
| 8 | Isorhamnetin | C_16_H_12_O_7_ | 316.06 | 5.63 | 315.05 |
| 9 | Naringenin | C_15_H_12_O_5_ | 272.07 | 6.09 | 271.06 |
| 10 | Pelargonidin | C_15_H_10_O_5_ | 270.05 | 6.15 | 271.06 |
| 11 | 4'-O-Glucosy lvitexin | C_27_H_30_O_15_ | 594.16 | 5.25 | 595.17 |
| 12 | Naringin | C_27_H_32_O_14_ | 580.18 | 5.29 | 579.17 |
| 13 | Orientin | C_21_H_20_O_11_ | 448.10 | 5.27 | 449.11 |
| 14 | Quercetin-3 -O-beta-glucopyranosyl  -6'-acetate | C_23_H_22_O_13_ | 506.11 | 5.48 | 505.10 |
| 15 | Quercetin-3β -D-glucoside | C_21_H_20_O_12_ | 464.10 | 5.47 | 463.09 |
| 16 | Rutin | C_27_H_30_O_16_ | 610.15 | 5.43 | 609.15 |

Note: RT represents retention time; m/z is the mass-to-charge ratio.

**Supplementary Table S7.** Coumarins in reed aqueous extracts

| **Number** | **Compound** | **Formula** | **Molecular Weight** | **RT [min]** | **m/z** |
| --- | --- | --- | --- | --- | --- |
| 1 | Coumarin | C_9_H_6_O_2_ | 146.04 | 3.98 | 147.04 |
| 2 | Esculetin | C_9_H_6_O_4_ | 178.03 | 5.29 | 177.02 |
| 3 | Esculin | C_15_H_16_O_9_ | 340.08 | 5.16 | 339.07 |
| 4 | Isofraxidin | C_11_H_10_O_5_ | 222.05 | 5.07 | 221.05 |

Note: RT represents retention time; m/z is the mass-to-charge ratio.
